# Determinants of stunting during the first 1,000 days of life in Bangladesh: A review

**DOI:** 10.1002/fsn3.1795

**Published:** 2020-07-20

**Authors:** Md Shariful Islam, Abu Naser Zafar Ullah, Shristi Mainali, Md. Akhter Imam, Md Imran Hasan

**Affiliations:** ^1^ Institute of Child and Mother Health Dhaka Bangladesh; ^2^ Public Health Daffodil International University Dhaka Bangladesh; ^3^ Marie Stopes Nepal Kathmandu Nepal; ^4^ Monitoring, Evaluation and Research Bangladesh National Nutrition Council Dhaka Bangladesh; ^5^ Center for Medical Research and Development Dhaka Bangladesh

**Keywords:** 1,000 days, Bangladesh, determinants, stunting

## Abstract

Stunting is a major problem in Bangladesh, with a prevalence of 31% in 2017. The prevalence of stunting in children aged under two has reduced by only 6% since 2004. After children reach 2 years of age, the consequences of stunting become almost irreversible. This paper seeks to examine and analyze the determinants associated with stunting during the first 1,000 days of life in Bangladesh to assist in developing evidence‐based interventions in Bangladesh. A literature review was conducted comprehensively on all relevant peer‐reviewed and gray literature of studies conducted in Bangladesh. The existing literature was searched and examined using the World Health Organization (WHO) conceptual framework for stunting. Evidence indicates that low maternal weight, lack of maternal education, severe food insecurity, lack of access to suitable nutrition, nonexclusive breastfeeding, pathogen‐specific diarrhea, and low weight and height at birth are associated with early childhood stunting in Bangladesh. The relation of the quality of drinking water with stunting is not clear in Bangladesh. Literature about the association between stunting and determinants such as the political economy, education systems, and agriculture and food systems is not found. This synthesis shows that the factors of stunting are multifaceted. As such, a multi‐sectoral approach is essential in Bangladesh, employing evidence‐based interventions to address the determinants that contribute to the risk of stunting to achieve the global nutrition target by 2025.

## Graphical abstract

Stunting is a major problem in Bangladesh, with a prevalence of 31% in 2017. Evidence shows that low maternal weight, lack of maternal education, severe food insecurity, lack of access to adequate nutrition, nonexclusive breastfeeding, pathogen‐specific diarrhea, and low weight and height at birth are correlated with early childhood stunting in Bangladesh. In Bangladesh, current studies are ambiguous about whether drinking water quality is correlated with stunting.

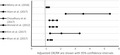

**Key messages**

- Evidence shows that low maternal weight, lack of maternal education, severe food insecurity, lack of access to adequate nutrition, nonexclusive breastfeeding, pathogen‐specific diarrhea, and low weight and height at birth are correlated with early childhood stunting in Bangladesh.
- In Bangladesh, current studies are ambiguous about whether drinking water quality is correlated with stunting.
- Literature about the prenatal factors of stunting is lacking in Bangladesh.

## 1 INTRODUCTION

There has been a considerable improvement in child health globally during the era of the Millennium Development Goals. Globally, the under‐five mortality rate decreased by half, from 90 per 1,000 live births in 1990 to 43 in 2015 (United Nations, 2016). Remarkable global progress was also made in reducing the prevalence of stunting among children under five, which fell from 47% in 1985 to 21.9% in 2018 (Stevens et al., 2012; UNICEF et al., 2019). However, progress toward a reduction in stunting remains poor in Bangladesh, where an average of 31% of children younger than five was stunted in 2017 (National Institute of Population Research and Training (NIPORT) & ICF, 2019).

A child is considered stunted if their height for age is more than two standard deviations below the median of the World Health Organization (WHO) Child Growth Standards (World Health Organization, 2014b). Stunting during the first 1,000 days of life is associated with both immediate and long‐term consequences, including reduced motor development, lower academic performance, and poor economic capability (Walker, Chang, Powell, & Grantham‐McGregor, 2005). Girls who were stunted in childhood not only remain shorter in stature in adulthood but also they tend to have stunted offspring (Black et al., 2013; Dewey & Begum, 2011).

The prevalence of stunting among children under five reduced by 15% between 2004 and 2014 in Bangladesh; however, only a 6% reduction in the prevalence was observed among children under 24 months over the period (NIPORT et al., 2016; NIPORT et al., 2005). Linear growth faltering often starts in utero. Most of the factors of stunting are usually developed or have a greater effect on children at an early age (<2 years) (Kuklina, Ramakrishnan, Stein, Barnhart, & Martorell, 2006). Stunting after the first 1,000 days of life is almost irreversible (Georgiadis & Penny, 2017). However, it is paradoxical that the reduction of the prevalence of stunting among children under two is low in Bangladesh. It is not clear whether the current nutritional approaches are aligned with the evidence in Bangladesh. The WHO also recommends that context‐specific factors should be considered to design actions and interventions to accelerate the rate of reduction (World Health Organization, 2014a). Hence, the identification of these context‐specific factors associated with stunting is a prerequisite in designing interventions to reduce it.

Several studies have identified the factors of stunting during the first 1,000 days in Bangladesh. A literature review is needed to analyze the evidence presented in the published literature and identify the current knowledge gap. For this reason, this review will examine and analyze the determinants associated with stunting during this vital stage of life in Bangladesh. This will help the government and development agencies to design better sustainable actions to reduce or prevent stunting during early life in Bangladesh, as well as in similar low–middle‐income countries.

## 2 METHODOLOGY

### 2.1 Search strategy

This literature review was conducted using five computerized bibliographic databases. To search peer‐reviewed literature, the PubMed, Cochrane Library, and Vrije University (VU) elibrary were used. Two databases, science.gov and BASE, were used to review gray literature. Both keywords and controlled vocabulary (when available) with combinations using Boolean operators like “OR” and “AND” were used. Two search attempts were performed to find literature using the five databases. The first attempt was a broad search to identify the relevant literature (Table 1). In the second attempt, we applied each element of the WHO conceptual framework for childhood stunting (see Figure 2). We further used the snowballing method to identify missed articles that met the inclusion criteria.

**TABLE 1:** Keywords and combinations in the two search attempts

First attempt					Second attempt				
AND					AND				
OR	Factor*	Stunt*	“1,000 day”	Bangladesh	OR	“Maternal Factors,” “Home environment,” “Complementary feeding,” Breastfeeding, Infection, “Political economy,” Health care, Education, Agriculture, Water, Flood Sanitation	Stunting	“1,000 day”	Bangladesh
	Cause	Undernutrition	Under‐two				Undernutrition	“24 months”	
	Determinants	“Linear growth retardation”	“24 months”				“Linear growth retardation”	Under‐two	
		Growth	Fetal				Growth	Fetal	
		IUGR	Infants				IUGR	Infants	

### 2.2 Inclusion and exclusion criteria

Articles covering fetal linear growth restriction and stunting among children under 24 months of age in Bangladesh were included. Furthermore, studies carried out in multiple countries were also examined when findings for Bangladesh were reported separately. Both peer‐reviewed and gray literature was included. Papers published in English and Bengali were included. To avoid compiling outdated results, articles published before 2004 were excluded.

### 2.3 Data extraction

**FIGURE 1:**
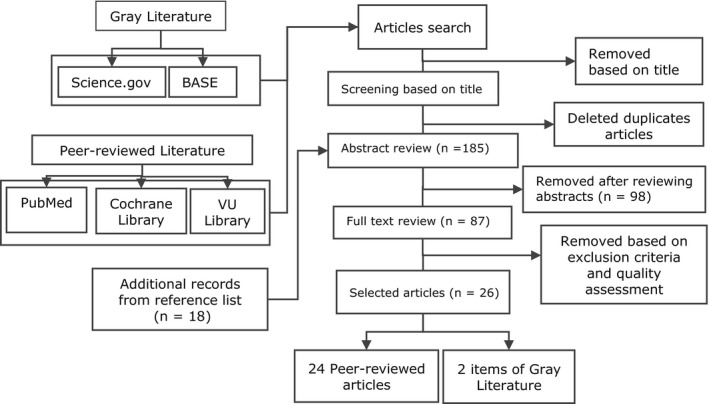
Flow chart of literature selection

We screened the title of articles after applying every search set mentioned in Table 1. If we found a relevant title, we retrieved the full article. We removed duplicate articles manually after collecting the full articles. Having reviewed the abstracts of 185 articles, we selected 87 for full‐text assessment based on the inclusion and exclusion criteria. We read the articles critically and assessed their quality. A total of 26 articles were selected for this review (details in Figure 1).

### 2.4 Quality assessment

The quality of the observational studies was assessed using the checklist of Strengthening the Reporting of Observational Studies in Epidemiology (STROBE) (Von Elm et al., 2014). A total of seven elements of quality appraisal criteria were used to assess the quality of articles, namely study design; effort to control bias; how the sample size was reached; methods of assessment; outcome measures; study limitations; and generalizability. We rated each study according to the seven criteria with scoring one point for each criterion they met. Papers had to score a minimum of four points to be selected. The quality of experimental studies was examined using four criteria, namely sample limitations of the study; inconsistency of results; bias; and possible confounders.

### 2.5 Conceptual framework

**FIGURE 2:**
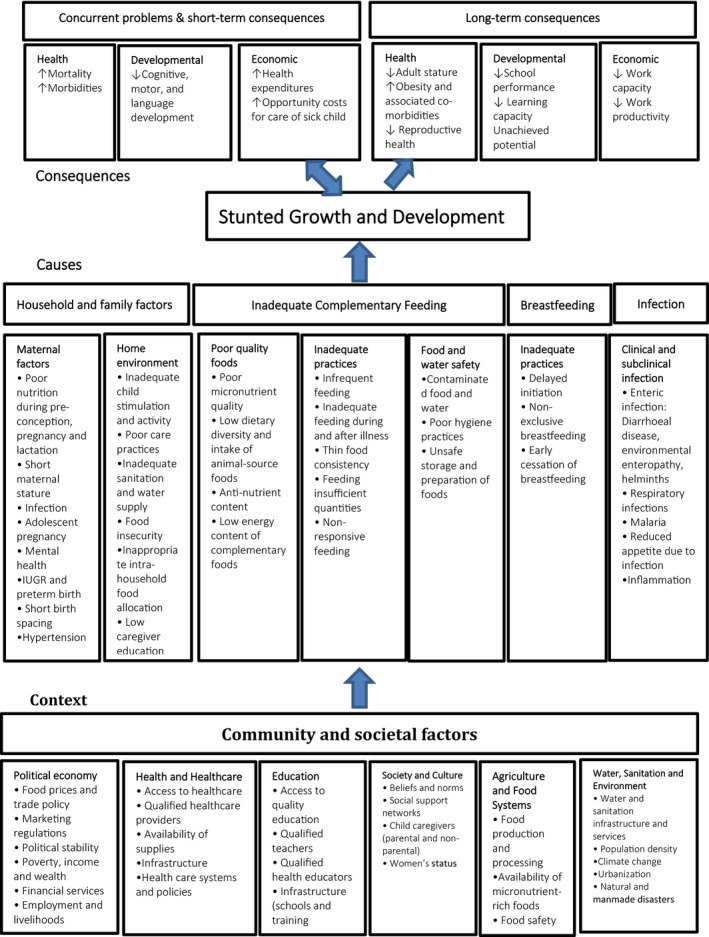
WHO conceptual framework for Childhood Stunting: Context, Causes, and Consequences (Stewart, Iannotti, Dewey, Michaelsen, & Onyango, 2013: P. 29)

The WHO conceptual framework for childhood stunting was used to guide this literature review (see Figure 2). The framework was prepared from global data. The causes section comprises four broad elements: households and family factors; inadequate complementary feeding; breastfeeding; and infection, with the latter including specific factors that may influence the growth faltering during the first 1,000 days of life. The contextual (community and societal) factors considered were political economy; health and health care; education; society and culture; agriculture and food; and water, sanitation, and environmental impact on the different underlying causes of childhood stunting.

## 3 RESULTS

### 3.1 Characteristics of selected studies

The selected studies were conducted in both urban and rural settings. Out of them, eight studies carried out in the urban area, 10 conducted in a rural setting, and eight studies covered both urban and rural areas. The majority of studies conducted from 2007 to 2016. The sample size varied a lot, ranged from 147 to 18,586. Out of them, 19 papers used primary data and seven analyzed existing sources of data. There are 10 cross‐sectional, three case–control, six cohort studies, and four randomized control trials.

### 3.2 Risk factors of early childhood stunting

#### 3.2.1 Maternal factors

WHO framework includes poor nutrition of the mothers, short stature, infection, adolescent pregnancy, mental health, IUGR, preterm birth, short birth spacing, and hypertension as maternal factors of stunting.

**FIGURE 3:**
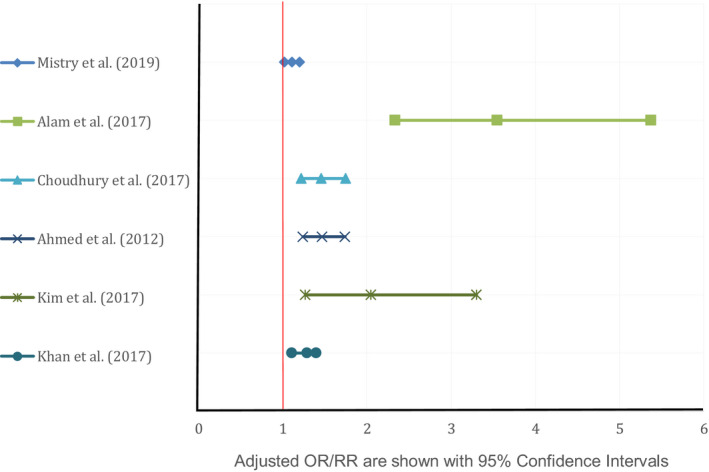
Adjusted OR/RR for underweight mothers found by different studies correlated with stunting

In total, eight studies investigated the influence of mother's nutrition on stunting among children aged 0–23 months in Bangladesh (Ahmed, Ahmed, Roy, Alam, & Hossain, 2012; Alam et al., 2017; Choudhury et al., 2017; Donowitz et al., 2018; Khan et al., 2017; Kim, Mejía‐Guevara, Corsi, Aguayo, & Subramanian, 2017; Mistry et al., 2019; Mondal et al., 2011). All reported a significant positive relationship between underweight mothers and poor linear growth of children (Figure 3). Donowitz et al. (2018) reported maternal weight at birth as the strongest predictor of linear growth at the age of 2 years. The children of underweight mothers (BMI < 18.5) had 1.11 (95% confidence interval, CI: 1.02–1.20) times the risk of being stunted than children of normal‐weighted mothers. In urban slum area, the odds of a mother with a BMI < 18.5 were 3.55 times higher (adjusted Odd Ratio, aOR 3.55, 95% CI: 2.34–5.38) among stunted children than nonstunted children (Alam et al., 2017). In a randomized control trial, Mridha et al. (2015) observed lipid‐based nutrient supplement during pregnancy and lactation reduced the risk of newborn stunting significantly (risk ratio, RR: 0.83; CI: 0.71–0.97).

Another strong determinant among children 0–2 years of age is the maternal short height (<145 cm) reported by Ahmed et al. (2012), Hasan et al. (2019), and Svefors (2018). The mothers with short height (<145 cm) had 4.7 times (95% CI: 2.28–9.56) higher chance of having a stunted child compared to mothers with higher height (Hasan et al., 2019). Goyal and Canning (2017) reported the children whose mothers age less than 18 years old were 1.15 times (95% CI: 1.08–1.122) higher risk being stunting. In Bangladesh, the adolescent pregnancy rate was 35% (Helen Keller International & BIGH, 2014). Black, Baqui, Zaman, Arifeen, and Black (2009) documented the odds of mothers' depressive symptoms were 2.17 times (95% CI: 1.24–3.81; *p* = .007) higher among stunted infants at 6–12 months than nonstunted infants in Bangladesh.

The correlation of maternal factors such as infection, hypertension, short birth spacing, and preterm baby with stunting of offspring was not examined due to lack of study in Bangladesh.

#### 3.2.2 Home environment

In the home environment subelement of the WHO framework, low caregiver education, inadequate child stimulation and activity, food insecurity, inadequate sanitation, and water supply, poor care practices, and inappropriate intrahousehold food allocation are mentioned as factors of stunting.

All of 10 studies reported a negative association between mother's education and stunting among the children aged 0–23 months (Table 2). The children whose mother had 10 or more years education had a 22% reduction in risk, and mother had 5–9 years education had a 12% decrease in risk being stunted than the children whose mother had no education (Mistry et al., 2019). However, only 12% of the mother had 10‐year formal education in 2013 (Choudhury et al., 2017). Svefors (2018) found father's education was also a protective factor for childhood linear growth faltering.

**TABLE 2:** Crude and adjusted odds/risk ratios with 95% CI for maternal education found by different studies correlated with offspring stunting

Articles	Crude OR		Adjusted OR		Characteristics
	OR	95% CI	OR	95% CI	
Choudhury et al. (2017)	4.06	3.18, 5.19	2.21	1.67, 2.92	No education
Hasan et al. (2019)	2.73	1.16, 6.42	2.05	0.82, 5.13	Illiterate
Raihan et al. (2018)	1.58	1.10, 2.26	1.03	0.67, 1.58	Never attend school
Ahmed et al. (2012)	_	_	0.47	0.34, 0.65	Education ≥ 10 years
Kim et al. (2017)	_	_	1.78	1.17, 2.70	Illiterate
Alam et al. (2017)	_	_	1.87	1.38, 2.54	Education < 5 years
Chakraborty (2009)	_	_	0.46	0.36, 0.59	Education ≥ 10 years
Svefors (2018)	_	_	1.74	1.17, 2.81	Education < 5 years
Mondal et al. (2011)	1.11	0.45, 2.71	_	_	Illiterate

Articles	Crude RR		Adjusted RR		Compared category	Reference category
	RR	95% CI	RR	95% CI		
Mistry et al. (2019)	0.61	0.52, 0.70	0.78	0.67, 0.92	Education ≥ 10 years	No education

The influence of household food insecurity on childhood stunting measured by five studies. Alam et al. (2017), Chakraborty (2009), and Mistry et al. (2019) documented a significant negative association between food insecurity and stunting among children under 2 years old. However, the other two studies that investigate according to the status of food insecurity, and they reported only severe food insecurity was responsible significantly for being short stature (Choudhury et al., 2017; Raihan et al., 2018).

Mistry et al. (2019) identified the quality of drinking water is not significantly associated with being short stature among children 0–2 years in Bangladesh. However, in the slum area, Alam et al. (2017) reported the odds of drinking untreated water were higher (aOR 1.51, 95% CI: 1.03–1.2.21) among stunted children than nonstunted children. In Bangladesh, 98% of the household had a safe water supply in 2017 (Bangladesh Bureau of Statistics, 2018). Improved and hygiene toilet were protective factors to prevent children from being stunted (Ahmed et al., 2012; Alam et al., 2017; Chakraborty, 2009; Mistry et al., 2019). The children who lived in a household with improved latrine were 12% (aRR 0.88, 95% CI: 0.79–0.98) less likely short stature that those living in a household with an unimproved latrine (Mistry et al., 2019).

The household factors include inadequate child stimulation and activity, poor care practices, inappropriate food allocation in the household were not assessed for association with child stunting in the literature in Bangladesh.

#### 3.2.3 Poor quality foods

This subelement of the WHO framework includes low dietary diversity and intake of animal source foods, poor micronutrient quality, antinutrient content, and low energy complementary feeding. No study found the association between antinutrient content in complementary feeding and stunting in Bangladesh.

In Bangladesh, only 30% of children 6–23 months of age received complementary feeding with ≥4 out of seven food groups in 2013. Low dietary diversity (less than four groups) in children was a risk factor of stunting (Choudhury et al., 2017). The children who received at least four out of six food groups, their height–age *Z* score was higher by 0.20 (*p* = .024) than the children who did not receive (Zongrone, Winskell, & Menon, 2012).

Christian et al. (2016) conducted a randomized control trial, reported prenatal multiple micronutrient supplementation reduced the prevalence of low length at birth (RR 0.95; 95% CI: 0.92–0.98). The effect of multiple micronutrients continued in the postnatal period, up to three months of age (RR 0.91; 95% CI: 0.88–0.94). Conducting a randomized control trial, Christian et al. (2015) observed supplementation of chickpea reduced the prevalence of stunting, 6.2% (95% CI: 10.6%–1.8%) at risk in the chickpea supplementation group than the control group.

#### 3.2.4 Inadequate feeding practices

Initiation of complementary feeding at or after seven months of age increased the risk of stunting by 1.23 times (adjusted *β* = 1.23, 95% CI: 1.05–1.44) than those started at age 5–6 months. However, complementary feeding before the age of five months was not associated (adjusted *β* = 1.25, 95% CI: 0.92–1.44) with chronic malnutrition (Owais et al., 2016). The children who took less than minimum frequent food, their risk being stunting was higher (Hasan et al., 2019; Owais et al., 2016). The children who received soft, semi‐solid, and solid food according to their age, the chance of chronic malnutrition was 1.34 times (*p* = .005) lower (Zongrone et al., 2012).

#### 3.2.5 Food and water safety

Hand washing of mother after child defecate (aOR 1.40, 95% CI: 1.02–1.93) and using the toilet (aOR 1.54, 95% CI: 1.08–2.21) are protective factors with children become stunting (Alam et al., 2017). Mistry et al. (2019) observed using soap after defecation and before eating of mother associated with offspring stunting and became insignificant in multivariate analysis in Bangladesh.

#### 3.2.6 Breastfeeding

Chakraborty (2009), Choudhury et al. (2017), and Mistry et al. (2019) documented the early initiation of breastfeeding (within 1 hr of birth) was not associated with childhood stunting. Chakraborty (2009) found the odds of initiating breastfeeding after 24 hr of birth were 1.21 times (aOR 1.21, 95% CI: 1.06–1.40) higher in stunted children than the counterpart. Chakraborty (2009) reported that the odds of receiving any food at <4 months and at 4–6 months of age were higher, 1.4 times (aOR 1.40, 95% CI: 1.20–1.60) and 1.18 times (aOR 1.18, 95% CI: 1.02–1.40) in stunted children than in nonstunted children, respectively.

#### 3.2.7 Infection

Schnee et al. (2018) identified the association between stunting and diarrhea was pathogen‐specific. The diarrhea caused by *Cryptosporidium, Campylobacter,* and *Shigella* associated with stunting in the first year of life and persisted up to 24 months, but not viral diarrhea (Schnee et al., 2018). The odds of *Cryptosporidium* infection had 2.69 times (aOR 2.69, 95% CI: 1.17–6.15) more in stunted children at 24 months, even this association existed nondiarrheal both asymptomatic and symptomatic infections. *Shigella* infection attributed to an average decrease of 0.24 cm (95% CI: 0.03–0.49) in height per episode per year (Schnee et al., 2018). The correlation of malaria, respiratory infection, and inflammation with stunting among children under 2 years of age was not studied in Bangladesh.

#### 3.2.8 Community and societal factors

Community and societal factors include political economy, health and health care, education, society, culture, agriculture, food systems, water, sanitation, and environment subelements in the WHO framework.

Seven studies investigated the influence of household wealth on childhood short stature and concluded a significant association (Table 3). Mistry et al. (2019) show the children living in the wealthiest household were 16% (aRR 0.84, 95% CI: 0.72–0.98) less likely stunted than those living in the poorest families.

**TABLE 3:** Crude and adjusted odds/risk ratios with 95% CI for household wealth quintile/ monthly income found by different studies correlated with offspring stunting

Articles	Crude OR		Adjusted OR		Characteristics
	OR	95% CI	OR	95% CI	
Choudhury et al. (2017)	1.76	1.44, 2.16	1.17	0.96, 1.44	Lowest
Islam et al. (2018)	2.95	1.49, 5.82	2.81	1.44, 5.52	Poor
Ahmed et al. (2012)			0.49	0.38, 0.63	Highest
Kim et al. (2017)			1.98	1.08, 3.63	Lowest
Chakraborty (2009)			0.45	0.37, 0.55	Highest

Articles	Crude RR		Adjusted RR		Compared category	Reference category
	RR	95% CI	RR	95% CI		
Mistry et al. (2019)	0.68	0.59, 0.79	0.84	0.72, 0.98	Highest	Lowest
Goyal and Canning (2017)			0.55	0.50, 0.6	Highest	Lowest

Antenatal (ANC) or postnatal care visit of mothers was a protective factor of childhood stunting (Choudhury et al., 2017). Chakraborty (2009) reported the odds of mothers with one ANC or no ANC visit having stunted child was 1.22 (aOR 1.22, 95% CI: 1.04, 1.44) and 1.32 (aOR 1.32, 95% CI: 1.15–1.50) times, respectively, than the odds of mothers with two or more ANC (Chakraborty, 2009). Chakraborty (2009) reported that the association between health care seeking for children from a qualified healthcare provider and stunting was not significant in Bangladesh. Mothers' exposure to any form (what so ever) of family violence increased the risk of children being short stature at birth, and this effect continued up to 24 months for both girls and boys in Bangladesh (Åsling‐Monemi, Naved, & Persson, 2009).

Temperature is associated with length at birth. The neonate born in the colder season was significantly shorter than those born in the summer season (Rashid et al., 2017; Svefors, 2018). Flood is one of the most common natural disasters in Bangladesh. Flood‐exposed children had around one‐inch lower height or about 0.2 less standard deviations than nonexposed children in Bangladesh (Del Ninno & Lundberg, 2005).

Mistry et al. (2019) found the children from Sylhet division (region) had 35% (aRR 1.35, 95% CI: 1.12–1.164) more chance being short stature than those from Dhaka division (region), while the risk was 16% (aRR 0.84%, 95% CI: 0.72–0.99) lower among children from Khulna division than Dhaka division.

The influence of the political economy, education quality, agriculture and food system, urbanization, climate change on childhood stunting in Bangladesh was not assessed due to lack of literature.

#### 3.2.9 Child characteristics

The WHO framework does not mention this subelement. However, we found low weight and length at birth, age, and being male was associated with stunting in Bangladesh.

Low weight and low length at birth were correlated with stunting child aged unde 2 years (Mondal et al., 2011; Nasreen, Kabir, Forsell, & Edhborg, 2013; Sanin et al., 2018; Svefors, 2018). On conditional random forest plot ranking, Svefors (2018) found height‐for‐age *Z* and weight‐for‐age *Z* score at birth were the most significant factors of stunting at 24 months of age.

**FIGURE 4:**
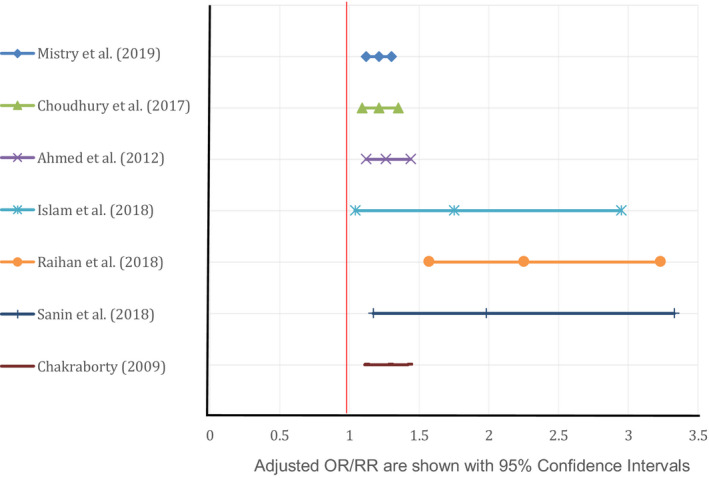
Adjusted OR/RR for being male found by different studies correlated with stunting

The relevance of gender with stunting investigated by nine studies in Bangladesh. Eight studies reported that the chance of being short stature was higher among boys than girls (Figure 4). Boys had a 21% (aRR 1.21, 95% CI: 1.12–1.30) higher risk being short stature than girls at an early age in Bangladesh (Mistry et al., 2019). Stratification of age shows the odds of boy age 6–11 months being stunting were higher than the odds of boys age 0–5 months or 12–23 months (Choudhury et al., 2017).

The association between the child's age and stunting was observed by six studies (Alam et al., 2017; Chakraborty, 2009; Choudhury et al., 2017; Islam et al., 2018; Mistry et al., 2019; Sanin et al., 2018). All identified child length growth faltering became significantly more common with the increase of age. The children 12–23 months of age were 2.65 (aRR 2.65, 95% CI: 2.20, 3.20) times more likely being stunting than 0–5 months old children in Bangladesh (Mistry et al., 2019).

## 4 DISCUSSION

To our knowledge, this is the first literature review that examines the determinants associated with stunting during the 1,000 days of life in Bangladesh.

In our analysis, mother's BMI < 18.5, height < 145 cm, age < 18 years, low education of mother, severe food insecurity, low diet dietary (<4 out of seven food groups), micronutrient adequacy, late initiating, less frequent, liquid and low energy content of complementary feeding, nonexclusive feeding, pathogen‐specific diarrhea are the causal factors stunting among children under 2 years of age in Bangladesh. Poor wealth status, family violence, flood, and resident in the Sylhet region are contextual factors that influence causal factors of stunting. Besides that, child characteristics such as low birth weight and length, being male and increasing age also correlated with stunting in Bangladesh.

The finding of this review is consistent with the finding of a systematic review conducted in Sub‐Africa (Akombi et al., 2017) and the result of a review performed in Indonesia (Beal, Tumilowicz, Sutrisna, Izwardy, & Neufeld, 2018). However, in this review, the insignificant association of the quality of drinking water, using soap after defection and before eating of the mother, healthcare‐seeking behavior with stunting was found which is inconsistent with the review mentioned above (Akombi et al., 2017; Beal et al., 2018). Rigorous research is needed in Bangladesh to understand and explain the association.

This review has several strengths. A comprehensive search of all existing articles was conducted on stunting in Bangladesh. The articles for this review were selected based on quality assessment, inclusion, and exclusion criteria. The finding, a specific association analyzed and compared using odds ratios, risk ratios, and 95% confidence interval. The selected articles were conducted in both urban and rural settings and using country representative data.

The WHO conceptual framework, we used in this review, was useful in determining a broad range of the factors that influence stunting during the first 1,000 days of life. We found child characteristics, such as age, sex, and low birth weight and length are also associated with early childhood stunting. However, this framework did not suggest these determinants. This recommendation can be added if multiple countries found the same finding.

The primary limitation of this study is that we did not apply meta‐analysis to understand the relationship between factors and stunting. It might be effective for a few determinants where heterogeneity observed. Another limitation is that the review, some studies conducted in the urban area, most of them were performed among the population with low‐socioeconomic status. The associated might be overestimated. Some studies investigated secondary data or survey data collected for different purposes. Caution is essential to interpret those findings. However, we included both peer‐reviewed articles and grey literature. Overall conclusions from this literature review demonstrate similar findings. We believe the analysis will be worthwhile for action planning on stunting and future research.

## 5 CONCLUSION

In this review, literature identified maternal undernutrition and education, severe food insecurity, poor wealth household, low birth weight, the biological factors age, and gender factors as common determinants of stunting among children age 0–23 months.

Several factors, maternal infection and hypertension, short birth spacing and preterm baby, inadequate child stimulation and activity, poor care practices, inappropriate food allocation in the household, anticontent content in complementary feeding, malaria, respiratory infection, and inflammation were not examined in this review due to lack of literature in Bangladesh. Among contextual factors, the political economy, education quality, agriculture and food system, urbanization, climate change, which were recognized in the WHO framework were not assessed in this review owing to the absence of studies. These knowledge gaps are needed to address by conducting research. We have analyzed some factors of linear growth retardation during the pregnancy period in this review. However, in prenatal linear growth restriction, many factors mentioned in the framework have not explored yet. More research required to understand the relationship between them.

Literature documented the quality of drinking water was not associated with childhood stunting in Bangladesh. Boys were dominantly stunted than girls; however, the cause is not still identified. Rigorous research needed to explore the association. Overall, the determinants of stunting are multi‐sectoral. Multi‐sectoral approaches will progress the stunting reduction rate in Bangladesh.

## CONFLICT OF INTEREST

We have no conflicts of interest.
